# The Effect of Wenxin Keli on the mRNA Expression Profile of Rabbits with Myocardial Infarction

**DOI:** 10.1155/2016/2352614

**Published:** 2016-10-24

**Authors:** Min Zheng, Zhouying Liu, Nana Liu, Cuihong Hou, Jielin Pu, Shu Zhang

**Affiliations:** State Key Laboratory of Cardiovascular Disease, Fuwai Hospital and Cardiovascular Institute, Chinese Academy of Medical Sciences and Peking Union Medical College, 167 Bei Li Shi Road, Xicheng District, Beijing 100037, China

## Abstract

*Aims*. The molecular mechanisms of Chinese traditional medicine Wenxin Keli (WXKL) were unknown. This study was aimed at exploring the effects of WXKL on the gene expression profile and pathological alteration of rabbits with myocardial infarction.* Methods*. Twenty male adult rabbits were randomly divided into 4 groups: sham, model, WXKL, and captopril groups. Model, WXKL, and captopril groups underwent the ligation of the left anterior descending coronary artery while sham group went through an identical procedure without ligation. WXKL (817 mg/kg/d), captopril (8 mg/kg/d), and distilled water (to model and sham groups) were administered orally to each group. After 4 weeks, the rabbits were examined with echocardiography and the hearts were taken for expression chip and pathological staining (H&E, Masson, and Tunel) studies.* Results*. The data revealed that WXKL downregulated genes associated with inflammation (CX3CR1, MRC1, and FPR1), apoptosis (CTSC and TTC5), and neurohumoral system (ACE and EDN1) and upregulated angiogenesis promoting genes such as RSPO3. Moreover, the results also showed that WXKL improved cardiac function and prevented histopathological injury and apoptosis.* Conclusion*. The present study demonstrated that WXKL might play an important role in inhibiting inflammation, renin-angiotensin system, and apoptosis. It might be a promising Chinese medicine in the treatment of patients with myocardial infarction.

## 1. Introduction

Coronary atherosclerotic heart disease (CAD) is a common disease usually caused by coronary stenosis and thrombus [1], among which acute myocardial infarction is much more emergent and dangerous in clinical practice. Acute myocardial infarction often leads to malignant arrhythmia or heart failure. Revascularization therapy such as percutaneous coronary intervention (PCI) has been proven to be an effective treatment for CAD; the modality is still high and the long-term prognosis is not satisfied [2].

Wenxin Keli (WXKL) is a Chinese herb extract composed of 5 agents and reported to be of great benefit in treating CAD, arrhythmia (atrial or ventricular), and heart failure [3]. It was found that WXKL could work by affecting action potential [4], blocking *I*_Ca-L_ [4], inhibiting *I*_to_ [5], and atrial-selectively depressing *I*_Na_ [6]. However, the effects of WXKL on acute myocardial infarction in molecular level are unknown. Therefore the current study was aimed at revealing the effects of WXKL on gene expression of myocardial infarction rabbit model and give new insights into the clinical application of WXKL in the setting of CAD.

## 2. Materials and Methods

### 2.1. Animals

Twenty male adult rabbits (*Oryctolagus cuniculus*) with body weight of 2-3 kg were randomly divided into 4 groups: the sham group (*n* = 5), the model group (*n* = 5), the WXKL group (*n* = 5), and the captopril group (*n* = 5). All groups except sham group underwent the surgery of ligation of the left anterior descending coronary artery while the sham group underwent an identical procedure without ligation. The procedure for care and treatment of animals all conformed to the Guiding Principles for Laboratory Animals issued by the Care of Experimental Animals Committee of the Chinese Academy of Medical Sciences and Peking Union Medical College.

### 2.2. Drugs and Reagents

WXKL was provided by Buchang Company, Xi'an, China, consisting of* Nardostachys* root and rhizome,* Codonopsis*,* notoginseng*, and amber. Captopril tablets were produced by Squibb Company, Shanghai, China. Before administration, WXKL and captopril were dissolved in distilled water.

### 2.3. Establishment of the Myocardial Infarction (MI) Rabbit Model

**Figure 1:**
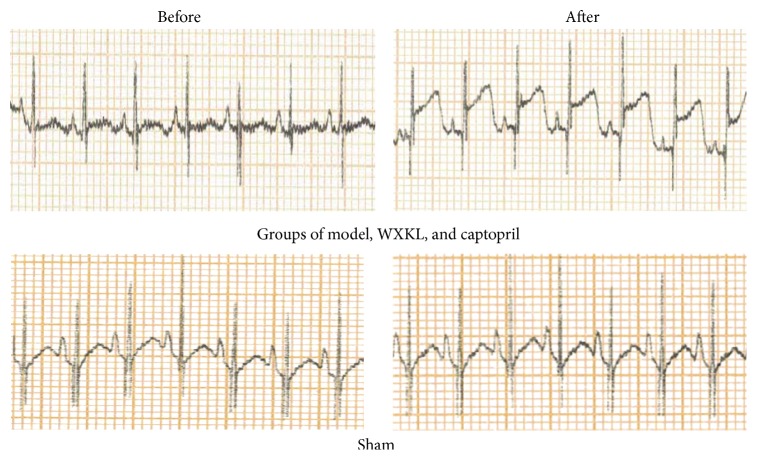
ECG (lead II) recorded during the operation. Ligation of the left anterior descending coronary artery was performed on model, WXKL, and captopril groups. ST segment elevation was recorded after the surgery (top). Sham group underwent thoracotomy but without ligation and ST segment showed no variation (bottom).

The rabbits were anaesthetized by ear vein injection of a 3% solution of sodium pentobarbital (30 mg/kg). During the surgery a series of procedures were performed as follows: endotracheal intubation, positive pressure ventilation, preoperative recording by twelve-lead electrocardiogram (ECG), one-lead monitoring, local skin disinfection, chest opening, thoracotomy device setup and opening of the pericardium, ligation of the left anterior descending coronary (2-3 mm from the bottom of aorta, usually between the left atrial appendage and pulmonary cone), closing of the pericardium, lung dilation, and chest closing. If the operation was successful, ST segment elevation was recorded by ECG (Figure 1) and the infarct myocardium became pale looking compared to other areas [7]. To prevent infection, 400,000 units of penicillin were injected within 24 hours after the surgery. In the sham group, the left anterior descending artery was not ligated. There was one rabbit that died of ventricular fibrillation 15 minutes after the ligation; the mortality rate was 6.25% during procedure. WXKL (817 mg/kg/d), captopril (8 mg/kg/d), and distilled water were given orally to their groups starting from the next day for 4 weeks.

### 2.4. Echocardiography Examination

Four weeks after the surgery, the noninvasive transthoracic echocardiography was used to evaluate the left ventricular structure and function after general anesthesia. In the two-dimensional ultrasound-guided M-curve, parameters of left ventricle like ejection fraction (EF), fractional shortening (FS), end-systolic dimension (ESD), end-diastolic dimension (EDD), end-systolic volume (ESV), end-diastolic volume (EDV), and end-systolic and end-diastolic thickness of left ventricular anterior wall (ESTLVAW and EDTLVAW) were recorded and analyzed by SPSS software to seek differences between different groups. After echocardiography evaluation, all rabbits were sacrificed.

### 2.5. RNA Extraction

One part of periphery tissue of the infarct zone was taken and washed in DEPC- (diethylpyrocarbonate-) treated water and frozen immediately in liquid nitrogen and then stored at −80°C until RNA extraction. Under the manufacturers' instructions, total RNA was extracted by mirVana mRNA isolation kit (Ambion, USA). Then, RNA quantity and integrity were assessed by NanoDrop ND-2000 (Thermo Scientific) and Agilent Bioanalyzer 2100 (Agilent Technologies), respectively.

### 2.6. Histopathology Examination

Another part of periphery tissue of the infarct zone was fixed in 4% formaldehyde and embedded in paraffin. Then Microtomes HM325 (Thermo Scientific, USA) was used to slice paraffin sections (5 *μ*m) which were intended to undergo H&E (hematoxylin-eosin), Masson, and Tunel (TdT-mediated dUTP Nick-End Labeling) staining. H&E staining was performed as the article described [8]. Masson and Tunel staining were conducted using Trichrome Stain (Masson) Kit (Sigma-Aldrich, USA) and In Situ Cell Death Detection Kit, POD (Roche, USA) separately. All procedures were according to the manufacturers' instructions. Microstructural changes were observed by light microscopy (Type BX51, Olympus, Japan) and photographed. Collagenous fibers were stained blue and nuclei of apoptotic cells were labeled positively by diaminobenzidine (DAB) as brownish yellow. The quantity of apoptotic cells was counted in 5 areas (×400) per group and the data were then averaged. The apoptosis rate was calculated by the following formula: apoptosis rate = number of apoptotic cells/number of nucleated cells.

### 2.7. Gene Expression Profiling

Three RNA samples of each group were randomly selected to conduct gene expression profiling. Double strand cDNA was transcribed by total RNA and synthesized into cRNAs which were then labeled with Cyanine-3-CTP. Labeled cRNAs were hybridized onto Agilent Rabbit Gene Expression Chip (4*∗*44K, Design ID: 020908, including 43,803 probes) based on manufacturer's instructions and scanned by Agilent Scanner G2505C (Agilent Technologies). Array images were processed by Feature Extraction software (version 10.7.1.1, Agilent Technologies) to get raw data. Genespring was used to conduct quantile normalization. According to fold change > 2.0 and *P* value ≤ 0.05, differentially expressed genes were selected by means of *t*-test. Gene ontology database and KEGG were applied to determine biological functions and signal pathways influenced by these differentially expressed mRNAs. Then, hierarchical clustering was performed to display the distinguishable genes expression pattern among samples.

### 2.8. Quantitative PCR

In order to confirm the outcomes of gene expression profiling, the interesting genes ACE, EDN1, CTSC, TTC5, MRC1, FPR1, CX3CR1, and RSPO3 were chosen to perform quantitative real-time reverse transcription-PCR (RT-PCR). LightCycler Probe Design software 2.0 (Roche Applied Bioscience) was used to design primers (Table 1). The reverse transcription reaction system consisted of 0.5 *μ*g total RNA, 2 *μ*L PrimeScript Buffer, 0.5 *μ*L oligo dT, 0.5 *μ*L random 6 mers, and 0.5 *μ*L PrimeScript RT Enzyme Mix I (TaKaRa, Japan) and cDNA was synthesized at 37°C for 15 min. Real-time RT-PCR reaction system contained 1 *μ*L of cDNA, 5 *μ*L 2x LightCycler 480 SYBR Green I master mix (Roche), 0.2 *μ*L forward primer, 0.2 *μ*L reverse primer, and 3.6 *μ*L nuclease-free water. All RT-PCR reactions were undergone using the LightCycler 480 II real-time PCR Instrument (Roche, Swiss) in triplicate under the following conditions: 95°C for 10 min, followed by 40 cycles of 10 s at 95°C, and 30 s at 60°C. To validate the specific generation of the expected PCR product, melting curve analysis was performed at the end of PCR cycles. The expression of each gene was normalized as ΔCt (Ct of target gene-Ct of internal control gene) using 18S rRNA as the control. The amounts of mRNA in model and WXKL groups were compared by the method of relative quantification using the ΔΔCt way.

### 2.9. Statistical Analysis

IBM SPSS Statistics (Version 21.0) was used to analyze the data. Echocardiography data were expressed as mean ± standard deviation (SD). One-way ANOVA was used to determine difference of basic parameters among groups of model, WXKL, and captopril. Independent sample *t*-test was used to compare data from groups of model, WXKL, and captopril to that from sham, respectively. *P* < 0.05 was considered statistically significant.

## 3. Results

### 3.1. Effects of WXKL on Cardiac Function

As shown in Table 2, the EF in model group was reduced significantly compared with that in sham group (49.23 ± 2.42% versus 65.30 ± 7.64%; *P* < 0.01). WXKL treatment significantly restored the EF; the averaged value was increased from 49.23 ± 2.42% to 57.10 ± 9.14% (*P* < 0.05) and had no statistical significance with captopril group (57.10 ± 9.14% versus 53.01 ± 9.14%, *P* > 0.05). The FS in the WXKL group was enhanced compared with that in model group (41.22 ± 7.42% versus 26.10 ± 2.68%, *P* < 0.01) but was unchanged compared with that in sham group (36.37 ± 7.14%, *P* > 0.05). These results suggested that WXKL could improve the cardiac function after acute myocardial infarction and the effect was not worse than or equivalent to captopril.

### 3.2. Effects of WXKL on Remodeling of Left Ventricle

As shown in Table 2, WXKL inhibited the remodeling of left ventricle after myocardial infarction; the mean values of ESD, ESV, EDD, and EDV in WXKL group were significantly reduced comparing with those in model group (all *P* < 0.05). These data, on the other hand, were not different between WXKL and sham groups (all *P* > 0.05). Apparently, WXKL improved the systolic and diastolic function of left ventricle and prevented left ventricular dilatation. Nevertheless, WXKL had no effect on ESTLVAW and EDTLVAW as seen in Table 2; the averaged data were similar in all four groups (all *P* > 0.05).

### 3.3. Effects of WXKL on Myocardial Histopathology

**Figure 2:**
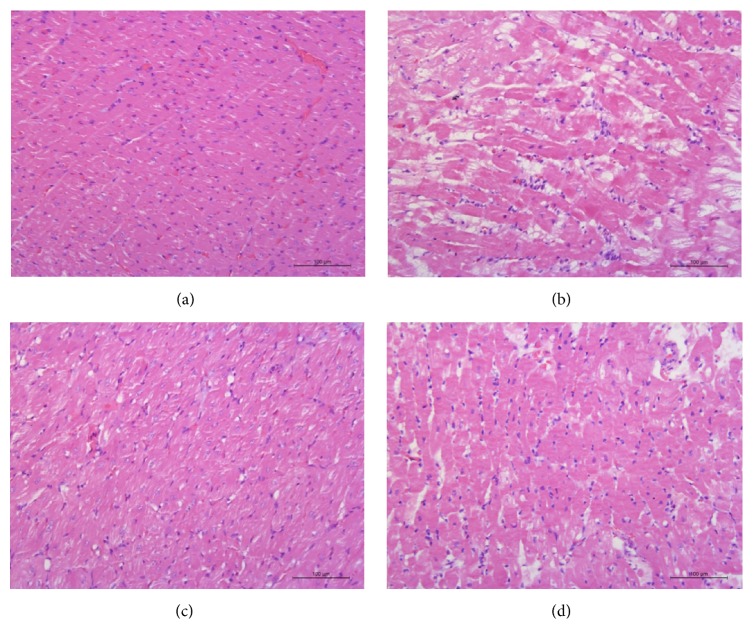
H&E staining (×400). Myocardial tissue slices were H&E stained and photographed by the digital camera connected with the optical microscope. (a) Sham group, (b) model group, (c) WXKL group, and (d) captopril group.

**Figure 3:**
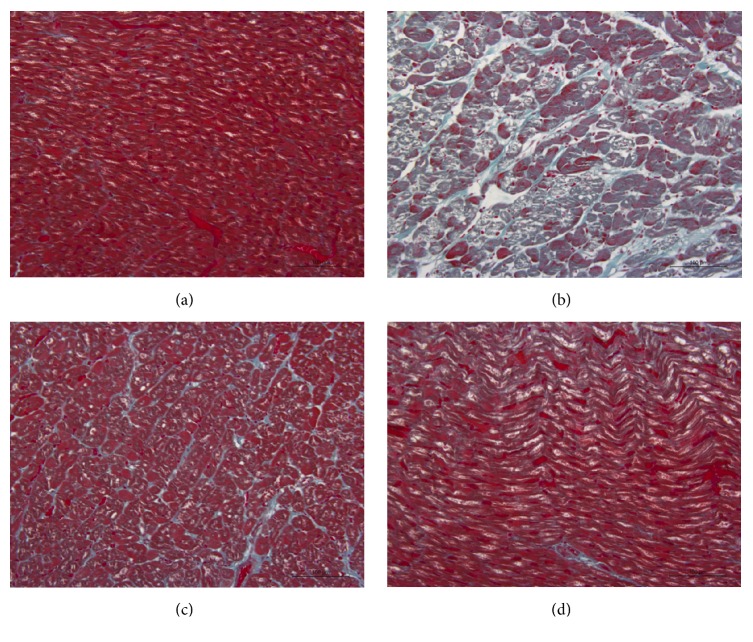
Detection of collagenous fibers by Masson staining (×400). Collagenous fibers were stained blue. (a) Sham group, (b) model group, (c) WXKL group, and (d) captopril group.

As shown in Figures 2 and 3, in sham group, cardiac cells were well arranged and cytoplasm was stained evenly with little collagen fibers. In contrast, the myocardium in model group showed interstitial edema, rich blue-stained collagen fibers, partial myocardial fiber necrosis and disorganized myocytes with unevenly stained cytoplasm, and numerous vacuoles. Many inflammatory cells (blue-stained nuclei) could also be seen gathered together in myocardium. However, the histopathological changes were significantly improved in WXKL and captopril groups, which suggested that the drugs might inhibit the inflammation and block the formation of collagen fibers.

### 3.4. Effects of WXKL on Myocardial Apoptosis

**Figure 4:**
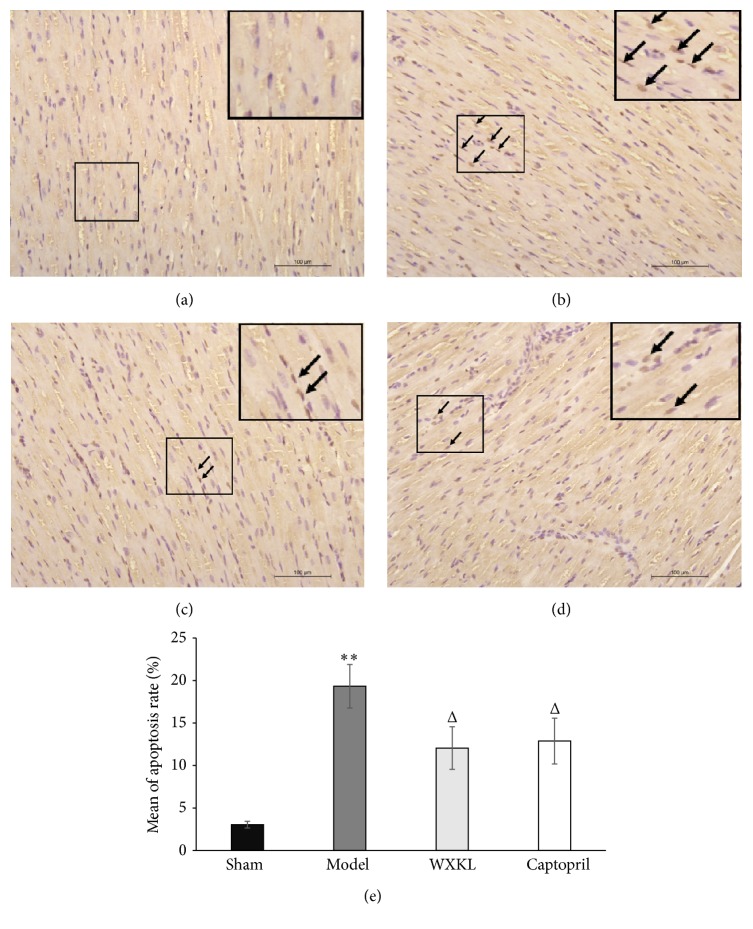
Detection of cardiomyocyte apoptosis with Tunel staining (×400). (a) Sham group, (b) model group, (c) WXKL group, (d) captopril group, and (e) quantitative analysis of apoptosis rates. As indicated by the arrows, nuclei of apoptotic cells were labeled as brownish yellow. Values were expressed as mean ± SD (*n* = 5). ^*∗∗*^ *P* < 0.01 versus sham. ^Δ^ *P* < 0.05 versus model.

As shown in Figure 4(a), in the sham group, nuclei were mainly stained blue with very few stained brownish yellow. By contrast, a lot of nuclei were stained brownish yellow in the model group, as seen in Figure 4(b). The number of apoptotic nuclei was significantly reduced in WXKL and captopril groups indicating the alleviated myocardial apoptosis, as shown in Figures 4(c) and 4(d). The apoptosis rates were calculated based on the mean quantity of apoptotic cells in each group. As shown in Figure 4(e), apoptosis rate in the model group was much higher (19.33 ± 2.56%) than that in WXKL group (12.05 ± 2.51%; *P* < 0.05) and captopril group (12.88 ± 2.69%; *P* < 0.05).

### 3.5. Effects of WXKL on Cardiac Gene Transcripts

**Figure 5:**
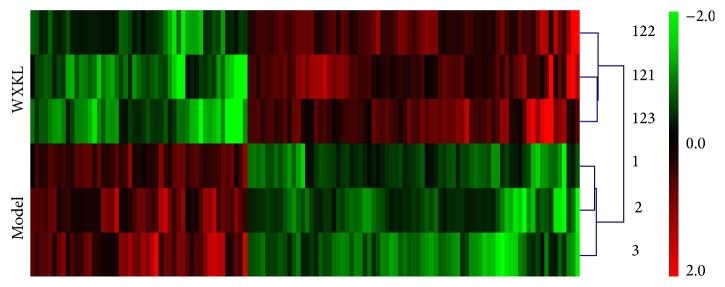
Hierarchical clustering of differentially expressed genes in model versus WXKL group by cluster. Gene expression profiles of WXKL effect on pathological rabbits were executed according to a 2-fold change cutoff. In total 126 genes were altered, among which 49 were downregulated and 77 were upregulated.

In order to determine the changes of gene expression pattern after acute myocardial infarction due to ligation of the left anterior descending coronary, gene expression profile was compared between sham and model groups and 229 genes in which the expression pattern was altered were identified, of which 114 were upregulated and 115 were downregulated. In order to further determine the effect of WXKL on expression profile, the differences between model and WXKL groups were compared and identified 126 genes in which the transcript levels were significantly changed. There were 77 genes that were upregulated and 49 that were downregulated. All these genes were displayed in Supplement Table  1 in Supplementary Material available online at http://dx.doi.org/10.1155/2016/2352614. In Figure 5 an overview of differently expressed genes was displayed between model and WXKL groups through hierarchical clustering analysis. In all of these differentially expressed genes, WXKL corrected 18 upregulated and 34 downregulated genes, so that there were a total of 52 genes restored by WXKL treatment (in Supplement Table  2).

After further analysis, it was found that WXKL affected genes were at least associated with immunity, inflammation, neurohumoral system, apoptosis, energy metabolism, and biosynthesis collagenous fiber development and none of these genes was associated with cardiac ion channels. Some of these genes were listed in Table 3. Our data revealed that WXKL downregulated a large number of immunity and inflammation related genes indicating that WXKL might play an anti-inflammatory role in the healing processes of myocardial infarction. This result was consistent with the findings that WXKL group showed less inflammatory cells infiltrating and less collagen fibers formation in the left ventricles than model group. Moreover, downregulated genes EDN1 and ACE were both important neurohumoral factors. Inhibition of these two genes could improve cardiac function and alleviate heart failure [9, 10], consistent with the findings that WXKL group showed better EF and FS than model group. Since CTSC and TTC5 could aggravate myocardial apoptosis [11, 12], their downregulation might reduce the number of apoptotic cells as it had been verified in the Tunel staining study that WXKL group had a lower apoptosis rate than model group.

Finally, comparing with captopril group, in WXKL group, there were 107 genes that were upexpressed and 69 genes that were downexpressed, as listed in Supplement Table  3. The majority of these genes were irrelevant to MI or heart functions; however, the voltage-gated potassium channel subfamily D member 2 (KCND2) might be important because it was of great importance in the myocardial restoration after MI. Besides, as shown in Supplement Table  3, there were a total of 188 different genes between WXKL and sham groups that had been identified, in which there were no interesting genes that directly related with remodeling of the heart after acute MI or heart failure.

### 3.6. Confirmation of Gene Expression by Quantitative Real-Time RT-PCR

**Figure 6:**
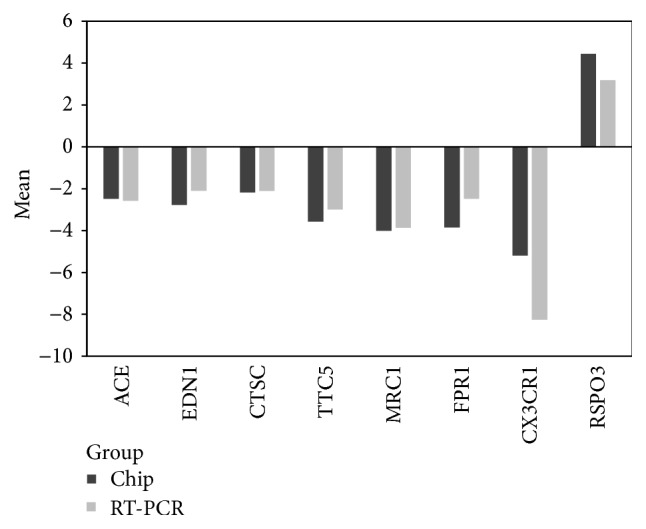
Confirmation of altered gene expression by quantitative real-time RT-PCR. ACE, EDN1, CTSC, TTC5, MRC1, FPR1, and CX3CR1 were downregulated while RSPO3 was upregulated in WXKL versus model group according to real-time RT-PCR. The relative mRNA expression level of each gene was normalized to 18S rRNA. The mRNA expression trends from real-time RT-PCR were in agreement with gene expression chip.

Eight genes (ACE, EDN1, CTSC, TTC5, MRC1, FPR1, CX3CR1, and RSPO3) were chosen to perform quantitative real-time RT-PCR to confirm the results from gene expression chip. Amplification quantity of ACE, EDN1, CTSC, TTC5, MRC1, FPR1, and CX3CR1 genes in WXKL group was lower and the RSPO3 gene was higher than that in model group. As displayed in Figure 6, the mRNA changes identified by real-time RT-PCR were consistent with that detected by gene expression chip.

## 4. Discussion

Myocardial infarction (MI) usually leads to decreased activity tolerance, reduced cardiac function (EF < 50%), heart failure, and so on due to acute loss of numerous myocardial cells [13, 14]. WXKL used to be an antiarrhythmic drug [5, 6] whereas previous studies showed that WXKL treatment for rats of MI model preserved cardiac function, reversed ventricular remodeling, and alleviated the histopathological damage [15]. The present study was aimed at investigating the molecular mechanism of WXKL by gene expression profiling. The results showed that WXKL had an influence on mRNA expression of genes involved in neurohumoral system, immune system, and cell apoptosis.

Studies demonstrate that neurohumoral activation plays an important role in the development of heart failure after myocardial infarction, including the renin-angiotensin-aldosterone system (RAAS) and the sympathetic nervous system [16]. Angiotensin II (AngII), which is subsequently cleaved from angiotensin I by angiotensin I converting enzyme (ACE), can activate multiple cardiovascular processes and lead to cardiomyocyte hypertrophy, ventricular remodeling, and myocardial fibrosis [17]. All these give rise to aggravation of heart failure with reduced ejection fraction [9]. Therefore angiotensin-converting enzyme inhibitor (such as captopril) is a standard therapy for patients with heart failure. According to the results of gene transcription profiling, WXKL downregulates ACE gene expression and reserves cardiac function after MI; the effects are similar to captopril.

In the meantime, WXKL reduces EDN1 (endothelin 1) gene transcription, which is a potent vasoconstrictor and has a key function in sympathetic innervation of the heart [10]. Increased concentrations of EDN1 are an extremely sensitive marker of heart failure severity [18] and predict a worse prognosis. Thus, reduced expression of EDN1 by WXKL could favor the recovery of cardiac function in the MI model.

In addition, unregeneration of cardiac muscle fiber gives rise to a reparative response like immune and inflammatory reaction, which will result in the formation of a scar and dilative remodeling of the ventricle [19]. Immunology and inflammation also work as pathogenic factors in the development and progression of chronic heart failure [19]. Immune activation may induce myocardial hypertrophy, contractile dysfunction, and myocardial cell apoptosis [14]. Recent studies show that anti-inflammation treatments may attenuate myocardial infarction and the subsequent ischemic heart failure [13]. Our data reveal that a large number of immune and inflammatory related genes are downregulated by WXKL (Table 3) including CX3CR1 (chemokine (C-X3-C motif) receptor 1), MRC1 (mannose receptor, C type 1), and FPR1 (formyl peptide receptor 1). All of these genes play an important role in triggering and maintenance of inflammation.

CX3CR1 (chemokine (C-X3-C motif) receptor 1) is highly expressed in human monocyte/macrophages and activates smooth muscle cells. It not only mediates patrolling of monocytes in the vascular space [9] but also regulates tissue macrophage functions. It is reported that CX3CR1 is also expressed in human atherosclerotic plaques [20]. Inhibition of CX3CR1 can reduce experimental arthrosclerosis in mice [21] and, conversely, gain-of-function polymorphism in CX3CR1 has been proved as a genetic risk factor of restenosis [22]. Thus downregulated CX3CR1 by WXKL may inhibit inflammatory response after myocardial infarction and reduce restenosis risk.

MRC1 (mannose receptor, C type 1), a scavenger receptor mainly expressed in the membrane of macrophages, assists macrophages in recognizing and binding the high-mannose structures of potentially pathogenic viruses and bacteria, mediating the endocytosis and causing immune response [23]. Moreover, FPR1 (formyl peptide receptor 1) which belongs to the family of formyl peptide receptors can be activated by N-formyl peptides [24]. Studies have demonstrated that FPR1 signaling pathway promotes the migration of innate immune cell and initiation of inflammation [25]. Hence, decrease of both genes by WXKL may, to some extent, play an anti-inflammatory role.

CTSC (Cathepsin C) and TTC5 (tetratricopeptide repeat domain 5) are both associated with cell apoptosis. CTSC is mainly responsible for activation of serine proteases in inflammatory and immune cells, which can trigger cell death in their target cells [11, 26]. On the other hand, TTC5 has been demonstrated to obviously activate p53-mediated cell apoptosis [12]. Since both of them are depressed by WXKL, this Chinese drug can mitigate apoptosis after myocardial infarction and the results are consistent with Tunel staining study.

Among upregulated genes by WXKL treatment, RSPO3 (R-spondin 3) is worth noticing. RSPO3 has been proved to promote angiogenesis in human endothelial cells, in favor of recovery after ischemic injury of heart [27]. Hence, we speculate that RSPO3 may also contribute to the restoration of the deteriorative cardiac function in WXKL group.

KCND2 (potassium voltage-gated channel subfamily D member 2), an Ito potassium channel encoding gene, is more increased in WXKL group than in captopril group. Ito plays a crucial role in controlling phase 1 of repolarization of the cardiac action potential (APD). Ito currents are always reduced after MI which leads to prolongation of APD and arrhythmia [28]. Therefore WXKL, but not captopril, may prevent arrhythmia after MI by increasing KCND2 gene expression.

## 5. Conclusion

WXKL effectively improves cardiac function and prevents from heart failure after acute myocardial infarction, probably by inhibiting neurohumoral system, suppressing inflammation, alleviating apoptosis, and stimulating angiogenesis. It may be a promising Chinese medicine in the treatment of acute myocardial infarction.

## Supplementary Material

Supplement Table 1 showed 229 differently expressed genes between sham and model groups, 126 altered genes between model and WXKL groups. Supplement Table 2 showed all the 52 genes restored by WXKL. Supplement Table 3 showed 176 differently expressed genes between WXKL and captoril groups, 188 changed gene between WXKL and sham groups.

## Figures and Tables

**Table 1:** Primers for quantitative real-time RT-PCR.

Gene symbol/GeneBank	Primer	Sequence (5′ → 3′)	Amplified length (bp)
ACE (NM_001171069.1)	Forward	AGACCTGGTCCAACATCTA	117
	Reverse	CAGCTTCCTCAAACATTCTC	

EDN1 (NM_001101696.1)	Forward	ACAACCGGACACATTGATGA	151
	Reverse	CCGGCTGGAAGAAGATACA	

CTSC (XM_002708644.2)	Forward	TGTAATGGTGGCTTCCCGTA	125
	Reverse	CGAAAGCAATCCTCCTTCA	

TTC5 (NM_138376.2)	Forward	CAGCTGGGTGAGGTGTACTG	125
	Reverse	AGCTGACGAAGCACCATTGA	

MRC1 (XM_002717356.2)	Forward	ACCTACTCAGACGGAGGTTA	108
	Reverse	AGAATCAGAAGGGTCACGATA	

FPR1 (NM_001082314.1)	Forward	CAGCAATGCCTCTCTTCC	107
	Reverse	CAAAGGTGACGACGAGTATC	

CX3CR1 (NM_001082134.1)	Forward	GAACCATCTTCCTGTCCATATT	133
	Reverse	GCCAGGTTGAGGAGGTAA	

RSPO3 (NM_032784.4)	Forward	CTGAAGGCCTCACCAGTGTT	100
	Reverse	GGTTAAGGTGGGTCATGCGA	

**Table 2:** Cardiac function, internal diameter, volume, and thickness of the left ventricle.

	Sham	Model	WXKL	Captopril
	(*n* = 5)	(*n* = 5)	(*n* = 5)	(*n* = 5)
EF (%)	65.30 ± 7.64	49.23 ± 2.42^*∗∗*^	57.10 ± 9.14^Δ^	53.01 ± 7.31^Δ^
FS (%)	36.37 ± 7.14	26.10 ± 2.68^*∗*^	41.22 ± 7.42^ΔΔ^	38.85 ± 3.09^Δ^
ESD (mm)	0.76 ± 0.33	1.30 ± 0.22^*∗*^	0.70 ± 0.39^ΔΔ^	0.67 ± 0.12^Δ^
EDD (mm)	1.16 ± 0.41	1.76 ± 0.24^*∗*^	1.29 ± 0.61^Δ^	0.97 ± 0.18^Δ^
ESV (mL)	0.66 ± 0.21	1.49 ± 0.25^*∗∗*^	1.01 ± 0.27^ΔΔ^	1.10 ± 0.17^Δ^
EDV (mL)	1.80 ± 0.73	2.93 ± 0.51^*∗*^	2.12 ± 0.93^Δ^	1.73 ± 0.26^Δ^
ESTLVAW (mm)	0.55 ± 0.17	0.43 ± 0.10	0.48 ± 0.13	0.40 ± 0.07
EDTLVAW (mm)	0.37 ± 0.12	0.34 ± 0.08	0.30 ± 0.08	0.26 ± 0.04

Data are expressed as mean ± SD.

EF, ejection fraction; FS, fractional shortening.

ESD, end-systolic dimension; EDD, end-diastolic dimension.

ESV, end-systolic volume; EDV, end-diastolic volume.

ESTLVAW, end-systolic thickness of left ventricular anterior wall.

EDTLVAW, end-diastolic thickness of left ventricular anterior wall.

^*∗*^ *P* < 0.05 versus sham. ^*∗∗*^ *P* < 0.01 versus sham.

^Δ^ *P* < 0.05 versus model. ^ΔΔ^ *P* < 0.01 versus model.

**Table 3:** Interesting genes associated with immunity and inflammation, neurohumoral system, angiogenesis, and apoptosis.

Effect on	Gene symbol	Gene name	Average log2 (fold change) in WXKL versus model	*P*
Immunity and inflammation	ACKR4	Atypical chemokine receptor 4	−2.0160573	0.041537866
	CLEC4A	C-type lectin domain family 4, member A	−3.1745374	0.010384568
	ICAM5	Intercellular adhesion molecule 5, telencephalin	−6.03934	0.03805122
	CX3CR1	Chemokine (C-X3-C motif) receptor 1	−5.196411	0.048417185
	MRC1	Mannose receptor, C type 1	−4.0125914	0.013496869
	LOC100349667	HLA class II histocompatibility antigen, DRB1-1 beta chain-like	−3.9618366	0.033953466
	FPR1	Formyl peptide receptor 1	−3.8557212	0.015122267
	RLA-DR-ALPHA	Major histocompatibility complex, class II, DR alpha	−3.5740817	0.038230587
	MS4A4A	Membrane-spanning 4-domains, subfamily A, member 4A	−3.4101653	0.015798943
	MSR1	Macrophage scavenger receptor 1	−2.8904014	0.026199685
	HCLS1	Hematopoietic cell-specific Lyn substrate 1	−2.644742	0.023556033
	RLA-DMB	Histocompatibility antigen DM heterodimer light chain-like	−2.438571	0.049889226
	LY86	Lymphocyte antigen 86	−2.4269795	0.037516456
	IGDCC4	Immunoglobulin superfamily, DCC subclass, member 4	3.3347745	0.014958037
	IGSF6	Immunoglobulin superfamily, member 6	−2.2142897	0.023191037

Neurohumoral factor	ACE	Angiotensin I converting enzyme	−2.4793751	0.037596557
	EDN1	Endothelin 1	−2.7699893	0.04192766
	CRYM	Myogenin (myogenic factor 4)	4.2102757	0.04186551
	UCN	Urocortin	2.0302756	0.021255666

Angiogenesis	RSPO3	R-spondin 3	4.436798	0.002975913

Apoptosis	CTSC	Cathepsin C	−2.184306	0.0108809
	TTC5	Tetratricopeptide repeat domain 5	−3.5752945	0.015227082

FC, fold change; *P*, *P* value calculated from two double factor variance analyses.
